# The high maternal TG level at early trimester was associated with the increased risk of LGA newborn in non-obesity pregnant women

**DOI:** 10.1186/s12944-018-0936-9

**Published:** 2018-12-26

**Authors:** Ning Liang, Haiyan Zhu, Xueping Cai, Zhiyin Le, Hongliang Wang, Dian He, Rong Xiao, Huanling Yu

**Affiliations:** 1 School of Public Health, Capital Medical University, Beijing, 100069 China; 2 FuXing Hospital, Capital Medical University, Beijing, 100069 China

**Keywords:** Triglyceride, Large for gestational age infant, Early pregnancy

## Abstract

**Introduction:**

Recent research had shown blood glucose was not the only cause of large for gestational age infant (LGA), the contributions of other fuels such as lipids also play an important role in fetal development. However the association between maternal triglyceride at early trimester and the risk of LGA has not yet been clearly elucidated. This research evaluated the association of maternal early trimester TG level with the risk of LGA infant in Chinese mothers.

**Methods:**

2839 pregnant women were recruited at the first visit of their perinatal health care and followed up prospectively till after delivery. The demographic, maternal characteristics were extracted from a questionnaire. Infant characteristics were collected at delivery. Maternal fasting serum total cholesterol (TC), triglyceride (TG), high density lipoprotein cholesterol (HDL-C), and low density lipoprotein cholesterol (HDL-C)levels, were measured in 6~8th, 16th, 24th, and 36th gestational weeks. Fasting serum glucose levels were measured at 6~8th, 24th, and 36th gestational weeks. Logistic regression model was used to calculate the odds ratio (OR) and 95% confidence intervals.

**Results:**

A consistently lower TG level was observed in mothers with non-LGA infant than mothers with LGA infant and TG level of mothers of LGA infants increased faster than that of control group. The incidence of LGA infants between two groups (TG<1.7 mmol/L and TG ≥ 1.7 mmol/L) was 14.46 and 26.63%, respectively. Mothers with the highest TG level (TG > 1.19 mmol/L) gave birth to infants with higher birth weight (BW) than the other two groups (TG < 0.70 mmol/L and TG:0.70~0.89 mmol/L). When stratified by pre-pregnancy body mass index (pre-BMI), a significantly positive association was founded between the maternal TG level at early trimester and the risk of LGA in non-overweight/obesity women (OR = 1.740, *p* = 0.034).

**Conclusions:**

The findings suggested that high maternal TG level at very early trimester was associated with the increased risk of LGA in non-overweight/obesity pregnant women. Moreover, high maternal TG level at first trimester may be an early predictor of LGA.

## Introduction

Fetal growth was affected by genetic, demographic and metabolic factors. Recent research showed that higher level of blood glucose was not the only cause of large for gestational age (LGA) [1]. The contribution of other fuels such as lipids to fetal birth weight has been investigated in many studies.

Serum lipid levels gradually increased from the 12th week of pregnancy, including total cholesterol (TC), triglycerides (TG), low-density lipoprotein-cholesterol (LDL-C), high density lipoprotein-cholesterol (HDL-C) and phospholipids, especially in the second and third trimesters [2–4]. These changes cannot reflect a pathological condition, but they represent a necessary adaptation of the mother’s physiology to satisfy the energy demand of mothers and the fetus as well as to prepare the maternal organism for delivery and lactation [5]. Previous research has indicated that maternal lipids play an important role during pregnancy, they are important for steroidogenesis of the mother, placenta and fetus, as well as for fetal growth [6]. But some studies had shown disturbances in maternal lipid metabolism could induce many kinds of adverse pregnancy outcomes (eg. preeclampsia, gestational diabetes mellitus, and preterm delivery) and moreover that can jeopardize short-term or long-term fetal health (eg. macrosomia, LGA, early cardiovascular disease, metabolic syndrome, insulin resistance) [7–9].

During whole gestational stages, high TG concentrations were associated with a raised risk for gestational impaired glucose tolerance (GIGT) and gestational diabetes mellitus (GDM) [10–12]. Previous research has indicated that maternal hyperlipidemia contributed to an increased morbidity of GDM and preeclampsia [13, 14]. It has been reported that TG level in third-trimester was associated with increased risk for LGA (OR = 1.13, 95% CI: 1.02–1.26) [7]. Another research also showed that high maternal TG level in third-trimester was significantly associated with LGA newborn [15].The Developmental Origin of Adult Diseases (DOHad) hypothesis proposes that maternal metabolic perturbations, and its subsequent intrauterine conditions, can program the fetus to be more prone for obesity. Maaike [16] suggested that both pre-BMI and maternal lipids during early pregnancy were independently related to offspring adiposity.

Many researchers had found that abnormal increase of serum TG level in the third trimester could induce macrosomia and LGA, but the association between early trimester maternal TG level and the risk for LGA still needed further confirmation. Therefore, the association between maternal TG level at early trimester and the risk of LGA in pregnant women was studied. The results might help to predict LGA infants in the early trimester.

## Methods

### Study sample

Pregnant women who were admitted to Fuxing Hospital affiliated to Capital Medical University and delivered at January 2016 to June 2017 were recruited for the present study. This study was established cohort based on following inclusion and exclusion criteria. The inclusion criteria were as follows: 1) singleton pregnancy; 2) naturally conceived. Women with pre-pregnancy cardiovascular disease, known diabetes, chronic hypertensive, and pregnancy-induced hypertension were excluded. Fetus with congenital malformation or APGAR scores<7 were also excluded.

Finally, 2839 antenatal cases accepted to take part in the study. Informed consent was gained at the initial visit. This study received ethical approval (2012SY29) from the ethics committee of Capital Medical University.

### Data collection and variables

The demographic, maternal characteristics were extracted from a questionnaire during their first perinatal health care, including maternal age, height, Pre-pregnancy weight, blood pressure, parity and history of disease (cardiovascular disease, diabetes, chronic hypertensive). Pre-pregnancy BMI (Pre-BMI) was calculated based on height and pre-pregnancy weight (BMI = weight/height^2^)and categorized into three levels using China cutoff points as underweight (BMI<18.5), normal weight (BMI<24.0 kg/m^2^) and overweight/obesity (≥24.0 kg/m^2^). Gestational weight gain (GWG) was calculated by subtracting the antenatal self-reported pre-pregnancy weight from the body weight at delivery.

According to the criterion of Association of Diabetes and Pregnancy Study Groups (IADPSG) which recommend a 75-gOGTT (Oral Glucose Tolerance Test) between 24~28 weeks of gestation, the diagnosis of GDM can be made when any one of the following values is met or exceeded in the75-g OGTT: 0 h (fasting), 5.10 mmol/L; 1 h, 10.00 mmol/L; and 2 h, 8.50 mmol/L.

Infant characteristics were collected at delivery, including gender, birth weight (BW), birth height, gestational age, delivery mode and perinatal outcomes. Gestational age was calculated based on combination of last menstrual period and the early first trimester ultrasound. Birth weight and height were measured in grams and centimeters. Newborns were classified into non-LGA (AGA and SGA), and LGA on the basis of International Fetal and Newborn Growth Consortium for the twenty-first Century (INTERGROWTH-21st) [3]. Neonates were defined as SGA if their birth weights fell below the 10th percentile for gestational age and as LGA if their birth weights exceeded the 90th percentile for gestational age. AGA was defined as birth weights met or exceeded the 10th percentile and met or fell below the 90th percentile for gestational age.

### Sampling

All participants were referred to do fasting blood tests at laboratory of Fuxing hospital, including serum total cholesterol (TC), triglycerides (TG), low-density lipoprotein-cholesterol (LDL-C), high density lipoprotein-cholesterol (HDL-C) and serum glucose. Fasting blood samples were drawn four times (at 6~8th, 16th, 24th, and 36th gestational weeks).

### Statistical analysis

All statistical analyses were performed using SPSS 21.0. The participants were divided into LGA and non-LGA groups based on the neonatal body weight. Normally distributed continuous variables were presented as mean ± standard deviation (SD), non-normally distributed continuous variables were presented as median (inter quartile range, IQR) and categorical variables were presented as N (%). Independent sample-test and Pearson’s chi-square test is respectively used to analyze the difference of continuous variables and categorical variables between two groups. In the multivariable adjusted model, maternal age, pre- BMI, GWG, parity, GDM were considered as confounding variables. *p* values < 0.05 were defined as statistically different.

## Results

### Maternal and child characteristics

Of the 2839 pregnant women, 435 delivered LGA infant. The prevalence of LGA in this research was 15.32%. Mothers with LGA infant were older than mothers in control group (31.30 ± 4.08 vs. 30.68 ± 3.70, *p* = 0.003). Mothers with LGA infant are also taller and heavier than mothers in control group (163.92 ± 5.08 vs. 162.61 ± 4.84, *p* < 0.001; 62.07 ± 9.71 vs. 57.67 ± 8.77, *p* < 0.001 respectively). As expected, pre-BMI and GWG of mothers delivering LGA infant were significantly higher than mothers in control group(23.05 ± 3.19 vs. 21.79 ± 3.04, *p* < 0.001; 13.72 ± 3.93 vs. 12.69 ± 4.14, *p* < 0.001 respectively). There are 27.59% mothers with LGA infant developed GDM which are significantly higher than 17.97% in control group (*p* < 0.001). Cesarean section was more frequent in mothers with LGA infant than in mothers with non-LGA infant (41.38% vs. 26.33%, *p* < 0.001). There was no statistically significant difference between the two groups in fetal gender (*p* = 0.230). All the results were shown in Table 1.

**Table 1:** Maternal and child characteristics

	Non – LGA *N* = 2405	LGA *N* = 435	*p* value^a^
Maternal characteristics			
Age (years)	30.68 ± 3.70	31.30 ± 4.08	0.003
Height (cm)	162.61 ± 4.84	163.92 ± 5.08	0.000
Weight (kg)	57.67 ± 8.77	62.07 ± 9.71	0.000
Pre-BMI(kg/m^2^)	21.79 ± 3.04	23.05 ± 3.19	0.000
Parity			0.000
1	1720(71.55)	251(57.70)	
>1	684(28.45)	184(42.30)	
Gestational weight gain (kg)	12.69 ± 4.14	13.72 ± 3.93	0.000
GDM			0.000
No	1972(82.03)	315(72.41)	
Yes	432(17.97)	120(27.59)	
Child characteristics			
Gender			0.230
male	1270(52.83)	216(49.66)	
female	1134(47.17)	219(50.34)	
Birth Height (cm)	49.26 ± 1.70	51.00 ± 1.41	0.000
Birth Weight (g)	3279.61 ± 344.94	3961.22 ± 290.10	0.000
Delivery mode			0.000
Vaginal delivery	1771(73.67)	255(58.62)	
Cesarean section	633(26.33)	180(41.38)	

All data are expressed as mean ± standard deviation, or number (percentage)

^a^Statistical significance of difference between non-LGA and LGA categories using the independent sample-test for continuous variables and the Pearson’s chi-square test for categorical variables

### Maternal TG level is associated with BW and the risk of LGA

**Fig. 1:**
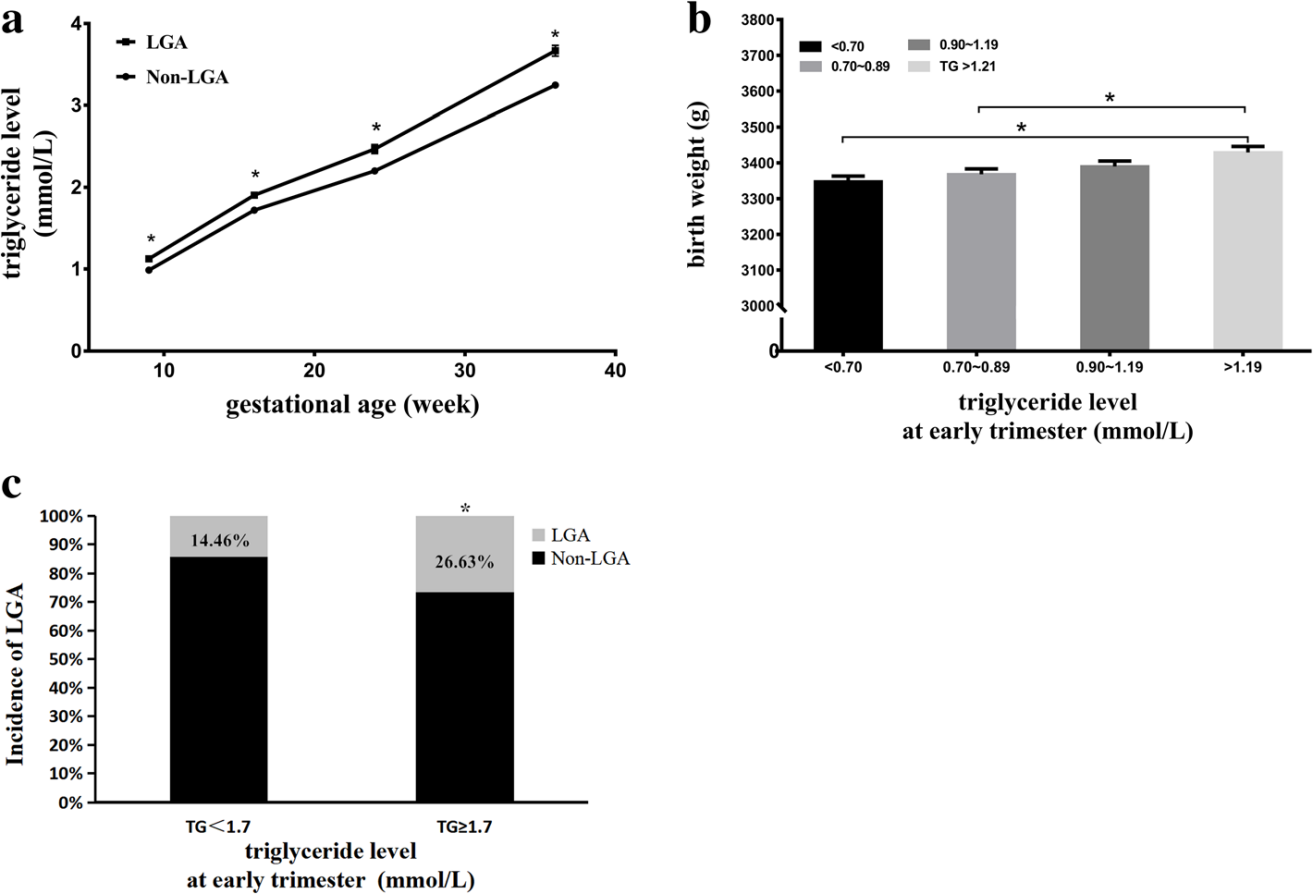
**a** Mean (±SEM) TG level between two groups. **b** TG level and Mean (±SEM) birth weight. **c** The incidence of LGA of different maternal TG level at early trimester .* *p* value < 0.05

Mothers with LGA infant had higher serum TG level. In analyses of longitudinal trends of TG level throughout pregnancy, a consistently lower TG level of non-LGA group than LGA group during whole gestation, and the TG level of mothers delivering LGA infant increased faster than control group (Fig. 1a). Mothers with the highest TG level (TG > 1.19 mmol/L) gave birth to infants with higher BW than the other two groups (TG < 0.70 mmol/L and TG:0.70~0.89 mmol/L) (Fig. 1b). The prevalence of LGA in the two groups (TG < 1.7 mmol/LandTG≥1.7 mmol/L) was 14.46 and 26.63% respectively, and there was a significant difference (Fig. 1c).

### Association between maternal TG level at early trimester and LGA

GWG, pre-BMI, parity, GDM and age were considered as confounding factors. Variables, including GWG, pre-BMI, parity, GDM and maternal TG level at early trimester were associated with the incidence of LGA. Maternal high TG level at early trimester (OR = 1.539, *p* = 0.021), GWG (OR = 1.105, *p* < 0.001), pre-BMI ≥ 24 (OR = 1.802, *p* < 0.001),parity>1 (OR = 1.890, *p* < 0.001) and GDM (OR = 1.729, *p* < 0.001) were positively associated with the incidence of LGA. Well pre-BMI<24 (OR = 0.413, *p* = 0.001) was a protective factor for LGA. Mothers with high TG level at early trimester (TG ≥ 1.7 mmol/L) experienced a 1.538 times increased risk of delivering LGA infant in comparison to the mothers with low TG levels (OR = 1.538, *p* = 0.013). All the results were shown in Table 2.

**Table 2:** The association between maternal TG level at early trimester and risk of LGA

Variables	OR	95% CI for OR	*p* value
TG(mmol/L)			
<1.7	Reference		
≥ 1.7	1.538	1.068–2.217	0.021
GWG	1.105	1.075–1.135	0.000
Pre-BMI			
18.5~23.9	Reference		
<18.5	0.413	0.246–0.694	0.001
≥ 24	1.802	1.404–2.314	0.000
Parity			
1	Reference		
>1	1.890	1.462–2.445	0.000
GDM			
No	Reference		
Yes	1.729	1.334–2.242	0.000

But when pre-BMI was stratified in two groups, maternal TG level at early trimester was positively associated with LGA (OR = 1.740, *p* = 0.034,) only in non-overweight/obesity women. All the results were shown in Table 3.

**Table 3:** The association between maternal TG level at early trimester and risk of LGA with pre-BMI

Variables	pre-BMI<24			pre-BMI ≥ 24		
	OR^a^	95% CI for OR	*p* value	OR^a^	95% CI for OR	*p* value
TG(mmol/L)						
<1.7	Reference			Reference		
≥ 1.7	1.740	1.042–2.908	0.034	1.410	0.840–2.366	0.193

^a^OR was adjusted by GWG, parity, GDM and age

## Discussion

With rapid economic growth in China during the past three decades, the improvement of living standards has brought about various problems of over-nourishment in pregnant women. Over-nourishment not only increased the incidence of LGA and macrosomia but also had many adverse effects on pregnant women and children health [17]. This research showed that high maternal TG level at early trimester was associated with the increased risk of LGA in non-overweight/obesity pregnant women.

Birth weight and incidence of macrosomia and LGA increased over the past four decades in many countries [18]. The incidence of LGA was 15.32% in this present population. In a research which observed secular trends of macrosomia in southeast China, the incidence of LGA births increased continuously from 13.72% in 1994 to 18.08% in 2000 [14]. The incidence of LGA was different in many cities in China, ranging from 8.2 to 17.7% [19, 20]. Facing such a severe epidemic trend, it is essential to reduce the incidence of LGA in China.

Maternal lipid level changed during normal pregnancy, generally beginning from 12th week of pregnancy [21]. So their concentrations at early trimester could reflect the pre-pregnancy lipid levels. Previous studies focused mainly on the effects of maternal mid-pregnancy or late pregnancy lipid profiles on birth weight of newborn [22, 23]. The present study was the first which emphasize the importance of maternal TG levels in very early pregnancy on delivering LGA infant before physiological gestational changes in its serum concentrations. This result could serve to reduce the incidence of LGA by controlling maternal TG level at early trimester.

From early pregnancy, maternal TG level rose constantly in both two groups (LGA and non-LGA), but a consistently higher TG level was observed in mothers delivering LGA infant than mothers delivering non-LGA infant. In addition, TG level of mothers delivering LGA infant increased faster than the control group. The women with high TG level (TG>1.21 mmol/L) are more likely to deliver big babies than women in the other two TG quintile, which indicated that maternal TG level at early trimester was associated with the birth weight. Vrijkotte [24] showed that mothers with higher TG level gave birth to infant with higher BW and mothers with the highest TG level(2.15 ± 0.52 mmol/L) gave birth to a significantly higher percentage of infants born LGA (12.9%) than women in the middle TG(1.23 ± 0.06 mmol/L)quintile(9.1%; *P* = 0.04). As expected, in this research LGA was developed in 26.63% of mothers with high TG level (TG ≥ 1.7 mmol/L) which is higher than 14.46% of control group. The findings suggested that maternal TG level at very early trimester was associated with birth weight and the incidence of LGA.

This study was in line with other researchers who reported positive associations of maternal TG level at early trimester and BW and LGA [24–26]. A research [25] showed the low TG level (<1.44 mmol/L) during early pregnancy was a protective factor for LGA (OR = 0.769). The results in this study showed that the early serum levels of TG increased the rate of delivering LGA infant. Mothers with high TG level (TG ≥ 1.7 mmol/L) were more likely to deliver LGA infant than mothers with low TG level (TG < 1.7 mmol/L). But when mothers were stratified in two groups (pre-BMI<24 and pre-BMI ≥ 24), significant difference was only found in mothers with normal weight, indicating the effect of pre-BMI which increased the risk of LGA may be stronger than TG level at early trimester. Consistent with this study, Vinod K. Misra [27] showed increased maternal serum TG was significantly associated with increased BW only for normal weight women at 10–14and 22–26 weeks gestation. But in research of Harmon KA [28], the 15th-week fasting TG correlated more strongly with infant adiposity than maternal obesity and 24-h glycemic level. Elaheh Mossayebi [29] had found that even in mothers with normal weight, there were 22.9% of mothers delivering LGA infant, and TG level was associated with the risk of LGA. Moreover, they also found that the effect of TG level was higher than fasting blood glucose on the incidence of macrosomia, but it should be noted that the TG level and fasting blood glucose were measured at the third trimester.

Until now the mechanism of hypertriglyceridemia affects fetal growth in pregnant women was poorly unknown. Recently, it was reported that lipids, specifical TG and free fatty acids (FFAs) may be strong contributors to LGA infant [30]. Therefore, it is very important to manage and control the maternal TG level during the first trimester in order to prevent the incidence of LGA and many other complicating diseases.

Maternal serum TG could not apparently transfer through the placenta before they were hydrolyzed into FFAs by placenta-specific hydrolase and lipoprotein lipase (LPL) [31]. Transplacental transfer of FFAs was important for fetal development, not only in energy supply but also in structural and metabolic functions [32]. Maternal TGs were hydrolyzed into FFAs in the placenta by endothelial-placental lipase, and maternal TG level could regulate the presence of these enzymes in the microvillous plasma membrane [33]. Therefore considering the physiologic mechanism of mothers with high TG level are more likely to deliver LGA infant, it could be hypothesized that hydrolysis of maternal TG by placental lipoprotein lipase to FFAs that crossing the placenta is increased [34].

On the other hand, FFAs had detrimental effects on the insulin resistance and impaired glucose tolerance [35]. Research had found enhanced insulin resistance during late pregnancy would explain the association between maternal TG level and fetal growth [33]. Insulin resistance promoted protein synthesis process and reduced lipolysis by accelerating rate of amino acid transfer system activation. In that way, high maternal TG level could result in macrosomia in the infant.

Placenta played an important role in fetal growth as all nutrients need to pass the syncytio-vascular layer of placenta to enter fetal circulation. TG could be hydrolyzed into FFAs and then those FFAs crossed the placenta and entered the fetus as important nutrients for fetal development [36, 37]. In addition, a research noted BMI and several of BMI-related metabolic factors (glucose, TGs, FFAs) on fetal fat accretion to a significant extent act by modifying placental weight [38]. Therefore, that mothers with high maternal TG level are more likely to deliver LGA infant may be the reason for the changes in placenta.

This study had some strengths and limitations. This research focused on the association between maternal TG level at early trimester and risk of LGA which was a novel perspective. In addition, this research had a large sample size. Because of its prospective design and the high rate of participation, information and selection bias were unlikely. The TG levels were measured at four time points and blood sampling was performed in a fasting state, which would not affect TG level. We did not collect information about the lifestyle of our participants such as dietary and physical activity factors which could act as confounders. On the other hand, this study cohort consisted of women who attended regular prenatal health care and intended to give a birth in Fuxing Hospital, which limits its results generalizability to the overall population and necessitates similar studies on other population.

Moreover, it should be noted that the maternal TG level at second and third trimester also can increase the risk of LGA. The effects of maternal TG level at second and third trimester were not investigated in this research and the independent contribution of maternal TG level at early trimester to fetal growth is still largely unknown. Deeper research is needed.

## Conclusions

In conclusion, high maternal TG level at early trimester was associated with the increased risk of LGA in non-overweight/obesity pregnant women. Moreover, high maternal TG level at first trimester may be the early predictor for LGA, which can help health care providers and obstetricians realize the importance and feasibility of controlling maternal TG level at the early trimester to decrease the morbidity of LGA and its related complications.

## Abbreviations

AGA: Aappropriate for gestational age
BMI: Body mass index
BW: Birth weight
DOHad: Developmental Origin of Adult Diseases
FFA: Free fatty acid
GDM: Gestational diabetes mellitus
GIGT: Gestational impaired glucose tolerance
GWG: Gestational weight gain
HDL-C: High density lipoprotein cholesterol
HDL-C: Low density lipoprotein cholesterol
IADPSG: Criterion of Association of Diabetes and Pregnancy Study Groups
IQR: Inter quartile range
LGA: Large for gestational age
OGTT: Oral glucose tolerance test
OR: Odds ratio
SD: Standard deviation
SGA: Small for gestational age
TC: Total cholesterol
TG: Triglyceride
